# Sub-types of safety behaviours and their effects on social anxiety disorder

**DOI:** 10.1371/journal.pone.0223165

**Published:** 2019-10-01

**Authors:** Emily Gray, Esther T. Beierl, David M. Clark

**Affiliations:** 1 Oxford Centre for Anxiety Disorders and Trauma, University of Oxford, Oxford, United Kingdom; 2 Oxford Health NHS Foundation Trust, Oxford, United Kingdom; Shinshu University School of Medicine, JAPAN

## Abstract

Cognitive models suggest that social anxiety disorder (SAD) is maintained through the use of safety behaviours. Previous reports propose that these safety behaviours can be subdivided into two main categories: avoidance and impression management. Study 1 investigates whether certain safety behaviours are specific to SAD. The social behaviour questionnaire was administered to individuals with SAD (*N* = 106), post-traumatic stress disorder (*N* = 28) and non-patient controls (*N* = 59). A factor analysis (*N* = 164) replicated the previously reported avoidance and impression management subtypes. Scores for both subtypes were significantly higher in individuals with SAD than in individuals with post-traumatic stress disorder or non-patient controls. Study 2 investigated the causal role of such safety behaviours using an experimental design in a non-clinical population (*N* = 96). Pairs of participants each engaged in two conversations. In one of the conversations, a randomly selected participant performed either avoidance or impression management safety behaviours. In the other conversation, neither participant was instructed to use safety behaviours. Each participant rated their own anxiety and performance as well as rating the other person. Videos of the conversations were also rated. Both types of safety behaviour increased anxiety in the person performing the safety behaviour. The avoidance subtype also had broader effects on the other person that were largely absent from the impression management subtype. Taken together the studies provide support for the distinction between safety behaviour subtypes and have implications for the treatment of SAD.

## Introduction

Social anxiety disorder (SAD) is a persistent fear of one or more social situations where embarrassment may occur and the anxiety is disproportional to the actual threat posed [1]. Individuals with SAD have distorted beliefs about how they are perceived by other people. In general, people with SAD come across more positively than they think [2, 3]. Given this, it is a puzzle why social anxiety persists, as logically an individual should adjust their beliefs of how they come across to others in response to the positive reactions they get from others. One of the reasons why individuals are thought not to adjust to a more realistic appraisal of themselves is that any positive outcomes are attributed to the use of social safety behaviours, rather than being a sign that the individual is intrinsically acceptable to other people [4].

Safety behaviours are overt or covert acts intended to prevent a feared outcome or to minimise its consequences [5]. They are found in many anxiety disorders, including post-traumatic stress disorder [6], obsessive compulsive disorder [7], panic disorder [8] and social anxiety disorder [9]. In SAD, social safety behaviours such as avoiding eye contact or rehearsing sentences before saying them are used in order to prevent feared outcomes such as public embarrassment or humiliation. However, as the fears of someone with SAD are excessive, it has been suggested that a major effect of safety behaviours is that they prevent patients from disconfirming some of their erroneous beliefs about this feared social situation [4]. Previous experimental evidence has provided support for the hypothesis that safety behaviours maintain social anxiety [4].

Several questionnaires have been developed to measure safety behaviours in social anxiety. The 28 item Social Behaviour Questionnaire (SBQ), [10, 11] was developed by Clark and colleagues in the 1990s provides the base for the studies detailed in this paper. The Subtle Avoidance Frequency Examination (SAFE), [12] developed by Cuming et al. consists of 32 items, which have been categorised as active safety behaviours, subtle restrictions of behaviour and behaviours aimed at concealing physical symptoms. The Social Phobia Safety Behaviours Scale (SPSBS), [13] was developed by Pinto-Gouveia, Cunha and Salvador and includes 17 items that partly overlap with those in the SBQ and the SAFE.

Several studies that used the SBQ have suggested that safety behaviours may fall into distinct categories which may not have identical effects. The first of these studies was conducted by Hirsch, Meynen and Clark [3], who divided the items in the SBQ into two groups based on face validity. The avoidance sub-group comprised behaviours that predominantly involved avoiding aspects of a social interaction (e.g. reduced eye contact, staying on the edge of a group, talking less). The impression management sub-group comprised behaviours which appeared to be attempts to closely monitor one’s performance and adjust it in order to try to convey a good impression (e.g. mentally rehearsing sentences, picturing how one is coming across). Hirsch et al. [3] found that the use of avoidance strategies in individuals with high levels of social anxiety was associated with poorer performance as judged by a conversational partner but impression management was not.

The second study examining sub-types of safety behaviour in social anxiety disorder was that of Plasencia, Alden and Taylor [14]. They performed two exploratory factor analyses on a version of the SBQ that was modified to make all items relevant to social interaction tasks. For this purpose, 11 of the 28 items were removed or combined and 2 items were added. One analysis was performed in a non-clinical undergraduate sample and one in a clinical sample of individuals diagnosed with SAD. In both analyses, Plasencia et al. found that safety behaviours fell into the two categories identified by Hirsch et al. [3]. Plasencia et al. [14] also found that when individuals with SAD were asked to have a conversation with a stooge, the self-reported use of avoidance safety behaviours negatively correlated with their partner’s wish to have a further interaction with the individual, whereas use of impression management safety behaviours was unrelated to partner responses.

Although Hirsch et al. [3] and Plasencia et al.’s [14] findings provide support for distinct subgroups of safety behaviours in social anxiety disorder which might have differential effects, there are several unanswered questions. First, as neither study included a control group of patients with another anxiety-related disorder, it is unclear whether social safety behaviours are specific to individuals with social anxiety disorder or are behaviours that are more broadly associated with anxiety *per se*. This is a critical issue as they can only be considered key maintaining factors for social anxiety disorder if they demonstrate some specificity to that condition. To address this issue, in Study 1 we compared scores on the SBQ in individuals with social anxiety disorder, individuals with another anxiety-related disorder (post-traumatic stress disorder) and non-patient controls. We also conducted a factor analysis within the social anxiety disorder patients and assessed the extent to which each identified subtype of safety behaviour was, or was not, specific to social anxiety disorder.

Second, the Hirsch et al. [3] and Plasencia et al. [14] studies are both correlational. The self-reported use of safety behaviours is correlated with adverse effects in social interactions, but it is unclear whether this represents a causal relationship. To more convincingly test for causation, it is necessary to experimentally manipulate the use of safety behaviours and observe their consequences. Study 2 includes such a manipulation.

## Study 1

### Uniqueness and subtypes

Study 1 comprised two parts. The first investigated whether the use of social safety behaviours is uniquely elevated in social anxiety disorder. The second aimed to replicate, in a clinical sample, the safety behaviour subtypes found by Plasencia et al. [14] in their factor analysis. Participants were individuals with social anxiety disorder, individuals with post-traumatic stress disorder and non-patient controls. The individuals with anxiety-related disorders were tested prior to the start of a course of cognitive therapy for their relevant condition at either the Oxford Centre for Anxiety Disorders and Trauma (OxCADAT) or the South London Centre for Anxiety Disorders and Trauma (CADAT). PTSD was deemed appropriate as a comparison to social anxiety disorder as it is a condition in which anxiety symptoms are prominent and the diagnostic manual at the time of data collection (DSM-IV) classified it as an anxiety disorder. The data was collected as part of a larger service audit into treatment for social anxiety disorder and PTSD. Individuals were assessed by trained clinicians using the Structured Clinical Interview for DSM-IV [15], for individuals with PTSD and with the Anxiety Disorders Interview Schedule for DSM-IV [16] for individuals with social anxiety disorder.

### Method

#### Participants

To investigate the specificity of safety behaviours, three groups of individuals were compared. The social anxiety disorder (SAD) group comprised 106 individuals who met DSM IV criteria for SAD. They were aged 20–56 (*M* = 33.27, *SD* = 8.38, 50.0% male). The post-traumatic stress disorder (PTSD) group comprised 28 individuals who met DSM IV criteria for that condition, a different anxiety-related disorder. They were aged 20–59 (*M* = 32.57, *SD* = 10.44, 35.7% male). The non-patient control group comprised 59 individuals, who did not meet criteria for any psychiatric disorder. They were aged 22–52 (*M* = 35.10, *SD* = 9.50, 42.4% male). For the factor analysis of safety behaviours, a larger group of 164 individuals was used, each of whom met DSM-IV criteria for SAD. This group included all those with SAD from the previous analyses plus individuals that were excluded from the previous analysis due to missing data on some of the required variables (such as FNE, BDI and BAI). They were aged 20–56, (*M* = 32.89, *SD* = 8.59, 48.9% male).

#### Materials

Each participant completed the social behaviours questionnaire (SBQ)[10], the Beck Depression Inventory (BDI) [17], the Beck Anxiety Index (BAI) [18] and the Fear of Negative Evaluation scale (FNE) [19]. All analyses in this paper were performed using IBM SPSS Statistics Version 23.0 [20].

*Social Behaviours Questionnaire*. The SBQ [10] is a 28 item questionnaire covering safety behaviours that could be used in social anxiety (e.g. try to come across well, try to control shaking). Participants are asked how often they use these behaviours in social situations and these are assigned a number (Never = 0, Sometimes = 1, Often = 2, Always = 3). The SBQ has been shown to have discriminant validity [10] and there was good internal consistency for this sample, α = .878. The SBQ can be downloaded from https://oxcadatresources.com.

*Fear of Negative Evaluation scale*. The FNE is a measure of fear of negative evaluation, a core part of social anxiety. It consists of 30 items with a true/false response format. It has been demonstrated to have high internal consistency, excellent test re-test reliability and to have discriminant validity [19].

*Beck Depression Inventory*. The BDI is a measure of depression. It comprises 21 items rated on a scale of 0–3, with total scores ranging from 0 to 63. The BDI has been shown to have good internal consistency, concurrent validity and test re-test reliability [21].

*Beck Anxiety Inventory*. The BAI is a measure of anxiety. Like the BDI, the BAI comprises 21 items rated on a scale of 0–3, with total scores ranging from 0 to 63. The BAI has been shown to be internally consistent, have good test re-test reliability and demonstrate good convergent and discriminant validity [22].

#### Statistical analysis

Hypotheses were tested using ANOVAs with post-hoc comparisons.

Subtypes of safety behaviours found in Plasencia et al. [14] were investigated using an exploratory factor analysis. The Kolmogorov-Smirnov test showed that the data was not normally distributed (*p* < .001 for all variables) and therefore principal axis factoring was used as the method of extraction, as recommended by Fabrigar, Wegener, MacCallum, and Strahan [23]. An oblique rotation was used (oblimin), because the factors were expected to correlate with each other, as they both assess safety behaviours in the broader sense. To determine the correct number of factors to retain, parallel analysis for a non-normal data set was used [24]. Parallel analysis produces random eigenvalues for the same number of variables and the same sample size, and from this it computes the number of factors whose eigenvalues are significantly higher than those produced by chance [21]. It is a highly recommended procedure as it produces appropriate numbers of extracted factors and reduces reliance on a researcher’s own interpretations [23, 25, 26]. A loading criterion of > .40 (as used by Plasencia et al. [14]) was used for item retention.

### Results

#### Characteristics of the groups

Between groups analyses were used to compare the three groups on measures of depression and anxiety as well as basic demographics. Table 1 shows means and standard deviations for the anxiety and depression measures. As expected, a one-way ANOVA showed a significant difference between the groups, with post-hoc comparisons indicating that patients with SAD score higher on the FNE than patients with PTSD and that both groups score higher than non-patient controls. By contrast, patients with SAD did not score higher than patients with PTSD on the general, non-social measure of anxiety (BAI) or on the measure of depression (BDI). Both groups scored higher than non-patients on each of these measures. The three groups did not differ in age (*F*(2,190) = 1.05, *p* = .353) or gender (χ^2^ (2, *N* = 200) = 2.39, *p* = .303).

**Table 1:** Differences in social anxiety (FNE), general anxiety (BAI), and depression (BDI) between SAD, PTSD and non-patient groups.

	Group			Statistic
Measure	Social Anxiety Disorder *M (SD)* *N* = 106	Post-Traumatic Stress Disorder *M (SD)* *N* = 28	Non-patient Controls *M (SD)* *N* = 59	
FNE	25.36 (5.27)^a^	16.63 (7.02)^b^	12.76 (6.66)^c^	*F*(2,190) = 89.81*
BAI	18.91 (8.84)^a^	20.73 (8.84)^a^	5.75 (6.33)^b^	*F*(2,190) = 56.99*
BDI	14.22 (8.01)^a^	16.34 (5.84)^a^	5.41 (5.28)^b^	*F*(2,190) = 36.99*

Note. BAI = Beck Anxiety Inventory, BDI = Beck Depression Inventory, FNE = Fear of Negative Evaluation scale.

* indicates p < .001.

Within each row, superscripts of the same letter indicate no significant difference between conditions, and superscripts of different letters indicate significant differences (p < .005).

#### Differences in safety behaviour use

A one-way ANOVA indicated that the three groups differed in mean SBQ scores (Table 2). Post-hoc comparisons showed that the SAD group scored significantly higher on the SBQ than the PTSD group, which in turn scored significantly higher than the non-patient controls.

**Table 2:** Means and standard deviations (in parentheses) for social safety behaviour use (SBQ) in SAD, PTSD, and non-patient groups.

	Group			Statistic
Measure	Social Anxiety Disorder *M (SD)* *N* = 106	Post-Traumatic Stress Disorder *M (SD)* *N* = 28	Non-patient Controls *M (SD)* *N* = 59	
SBQ	1.43 (.32)^a^	1.03 (.37)^b^	0.77(.31)^c^	*F*(2,192) = 83.76*

SBQ = Safety Behaviour Questionnaire.

* indicates p < .001.

Within each row, superscripts of the same letter indicate no significant difference between conditions, and superscripts of different letters indicate significant differences (p < .005).

#### Exploratory factor analysis

**Fig 1:**
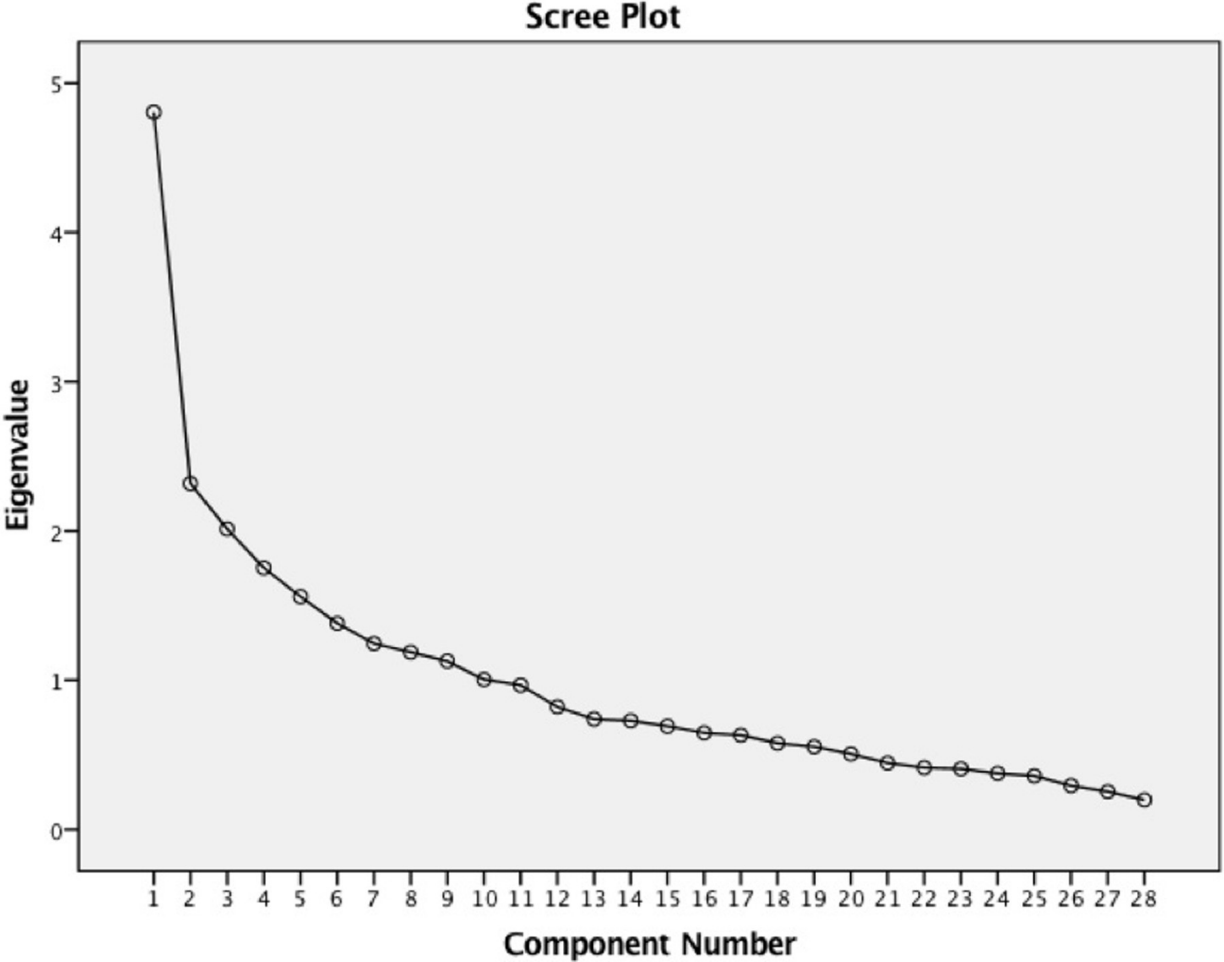
Scree plot showing the eigenvalues of extracted components of the SBQ data.

To identify subtypes of safety behaviour, an exploratory factor analysis was performed on the 28 items of the SBQ as completed by individuals with SAD (*N* = 164, *M* = 1.45, *SD* = .31). The Kaiser-Meyer-Olkin measure of sampling adequacy (KMO = .71) and Bartlett’s test of sphericity (χ^2^(378) = 1164.63, *p* < .001) indicated that the data was suitable for factor analysis as variables were intercorrelated. The parallel analysis showed that two factors were appropriate for extraction. Together the two factors explained 25.79% of the variance with the first factor explaining 17.26% (*λ* = 4.83) and the second explaining 8.53% (*λ* = 2.39). Fig 1 shows the scree plot for this extraction.

The pattern matrix of the two-factor solution was examined. Table 3 shows all items and their standardized factor loadings.

**Table 3:** Loadings of SBQ items onto the two extracted factors.

Item	Loading on factor 1: Avoidance	Loading on factor 2: Impression Management
1- Use alcohol to manage anxiety	-.04	.22
2- Try not to attract attention	**.45**	.12
3- Make an effort to get your words right	.27	.39
4- Check that you are coming across well	.04	**.64**
5- Avoid eye contact	.25	-.09
6- Talk less	**.68**	-.11
7- Avoid asking questions	**.51**	-.09
8- Try to picture how you appear to others	.16	**.61**
9- Grip cups or glasses tightly	.10	.33
10- Position yourself so as not to be noticed	**.51**	.15
11- Try to control shaking	.01	.18
12- Choose clothes that will prevent or conceal sweating	.00	.18
13- Wear clothes or makeup to hide blushing	.02	.32
14- Rehearse sentences in your mind	.38	.23
15- Censor what you are going to say	**.44**	.35
16- Blank out or switch off mentally	.20	.17
17- Avoid talking about yourself	**.46**	.14
18- Keep still	**.52**	.04
19- Ask lots of questions	-.20	.36
20- Think positive	.17	.04
21- Stay on the edges of groups	**.50**	.00
22- Avoid pauses in speech	.20	.10
23- Hide your face	.16	.24
24- Try to think about other things	.12	-.13
25- Talk more	-.39	.26
26- Try to act normal	-.10	**.49**
27- Try to keep tight control of your behaviour	.05	**.55**
28- Make an effort to come across well	-.16	**.63**

Note: Bold font indicates item retention on the relevant factor.

The avoidance factor (factor 1) includes eight behaviours. In order of loading strength these are: ‘Talk less‘ (Item 6) ‘Keep still’ (Item 18), ‘Avoid asking questions’ (Item 7), ‘Position yourself not to be noticed’ (Item 10), ‘Stay on the edge of groups’ (Item 21), ‘Avoid talking about yourself’ (Item 17), ‘Try not to attract attention’ (Item 2), and ‘Censor what you are going to say’ (Item 15).

The impression management factor (factor 2) includes five behaviours. In order of loading strength, these are: ‘Check that you are coming across well’ (Item 4), ‘Make an effort to come across well’ (Item 28), ‘Try to picture how you appear to others’ (Item 8), ‘Try to keep control of your behaviour’ (Item 27), and ‘Try to act normal’ (Item 26).

Differences in loadings between the factors are generally moderate to large, and the correlation between the factors is moderate (*r* = 0.39, *p* < .001), suggesting a clear differentiation of the avoidance and impression management factors. The two factors clearly fall into the conceptually defined groups identified by Hirsch et al. [3] and explored in Plasencia et al.’s [14] factor analysis.

**Are both subtypes of safety behaviour uniquely elevated in social anxiety disorder?**. Following the extraction of the subtypes, separate sub-scores were created for each individual. This was done by calculating an individual’s mean score on the five items that significantly loaded onto the impression management factor and the eight items that significantly loaded onto the avoidance factor. The two sub-scores had acceptable internal consistency (factor 1; α = .76; factor 2; α = .75) for individuals with social anxiety.

One way analyses of variance were used to compare the sub-scores in the different groups of participants (Table 4). These were significant for both avoidance and impression management safety behaviours. Post hoc comparisons indicated that patients with SAD scored significantly higher than patients with PTSD on both impression management and avoidance safety behaviours. The PTSD group in turn did not differ to the non-patient controls on impression management (*p* = .14) but scored higher than non-patients on avoidance (*p* < .05).

**Table 4:** Differences between groups in the use of impression management and avoidance safety behaviours.

	Mean (SD)			Statistic
Social behaviour subtype	SAD *N* = 106	PTSD *N* = 28	Non-patient controls *N* = 59	
Impression management	1.90^*a*^ (.58)	1.44^*b*^ (.57)	1.18^*b*^ (.57)	*F*(2,192) = 31.19*
Avoidance	1.62^*a*^ (.51)	1.01^*b*^ (.53)	.74^*c*^ (.40)	*F*(2,192) = 67.10*

* indicates p < .001.

Within each row, superscripts of the same letter indicate no significant difference between conditions, and superscripts of different letters indicate significant differences (p < .05).

### Discussion

The first aim of the study was to determine whether social safety behaviours are specifically elevated in social anxiety disorder or are a function of anxiety *per se*. By including a control group of patients with PTSD who scored as high as patients with SAD on a general measure of anxiety (BAI) and depression (BDI), we were able to distinguish between the unique effects of social anxiety and those of general anxiety or depression. Our findings indicate that social safety behaviours are especially elevated in social anxiety disorder.

A second aim of the study was to investigate whether the distinction between avoidance and impression management safety behaviours that emerged in Plasencia et al.’s [14] factor analysis could be replicated in a new sample of patients with social anxiety disorder. Our findings generally replicate those of Plasencia et al. [14], showing that the distinction between avoidance and impression management safety behaviours is factorially valid.

A final aim was to determine whether both types of safety behaviour are especially elevated in SAD. We found that patients with SAD reported greater use of both types of safety behaviour than patients with PTSD, confirming that each subtype is particularly elevated in SAD. Patients with PTSD did not differ from non-patients in their use of impression management safety behaviours. However, they did report being more likely to use avoidance safety behaviours than non-patients. The increase in the use of avoidance safety behaviours in PTSD may have a different motivation to their more extensive use in SAD. In particular, they may reflect the tendency of individuals with PTSD to avoid talking to other people about their trauma. This tendency could explain why PTSD patients report more avoidance behaviours such as saying little and staying on the edge of groups when compared with non-patients.

The amount of variance in SBQ scores accounted for by the avoidance and impression management factors was less in our study (26%) than in Plasencia et al.’s [14] study (44%), although the number and type of SBQ items that loaded on each factor was very similar. Plasencia et al.’s [14] factor analysis probably accounted for more SBQ variance because they dropped 11 items from the questionnaire and we found that most of the dropped items failed to load either factor. However, as these items also discriminated between patients with social anxiety disorder and PTSD, it seems that a fully comprehensive account of safety behaviours in social anxiety needs to go beyond the two factors studied here. Cumming et al. [12] identified a factor concerned with attempts to hide the physical symptoms of anxiety which is also likely to be important. The SBQ “Blank out or switch off mentally” and “Try to think about other things” suggest that a further factor linked to certain mental operations may be important.

## Study 2

Study 2 experimentally manipulated the use of avoidance and impression management safety behaviours during a conversation in order to determine whether they had a causal role in generating anxiety and other adverse effects. The study was approved by the Oxford University Medical Sciences Inter-divisional Research Ethics Committee (Ref: MS-IDREC-C1-2015-199).

Pairs of participants (dyads) each engaged in two conversations. In one of the conversations, a randomly selected participant performed either avoidance or impression management safety behaviours. In the other conversation, neither participant was instructed to use safety behaviours. Each participant rated their own anxiety and performance as well as rating the other person. Videos of the performance were also rated.

We assumed that any adverse effects of safety behaviours would be observable in all individuals who perform such behaviours, rather than simply an effect that can only be observed in individuals with social anxiety disorder. Consistent with this assumption, the experiment was conducted in a non-clinical sample.

### Method

#### Design

Participants performed the experiment in pairs (dyads). Each pair had two, five-minute conversations. In one of the conversations, one participant was instructed to perform safety behaviours (the experimental participant) and one was not (the control participant). These safety behaviours were either avoidance or impression management behaviours. In the other conversation, neither participant performed safety behaviours. Whether the safety behaviours were performed in the first or second conversation was counterbalanced across participants and within each safety behaviour subtype.

After each conversation, participants were asked to rate how anxious they felt, how anxious they thought they appeared, how anxious they thought their partner looked, how much they liked their partner, how much they enjoyed the conversation, and the extent to which they were self or externally focused. After both conversations, the participants were also asked how much they would like to have a similar conversation again. Later, the experimenter, who was blind to condition, rated each participant’s performance from video recordings of the conversations.

#### Participants

Ninety-six undergraduate students took part in this study. The mean age was 20.80 years (*SD* = 3.72) and 58 were female. Participants were required to score below 20 on the BDI and below 2 on question 9 (suicidal ideation) [13], and not be currently receiving treatment for mental health issues or have ever been diagnosed with social anxiety disorder. These exclusion criteria, which were required by the local ethics committee, were intended to ensure that a non-clinical population was selected and potentially vulnerable individuals did not participate.

#### Materials

Participants were screened online using two questionnaires. The BDI [17], to screen for depression, and the Albany Panic and Phobia Questionnaire, Social Phobia Subscale (APPQSP) [27], which was used to ensure that participants did not vary in levels of social anxiety between experimental conditions.

*Beck Depression Inventory*. As detailed above, the BDI is a measure of depression. It comprises 21 items rated on a scale of 0–3, with total scores ranging from 0 to 63. The BDI has been shown to have good internal consistency, concurrent validity and test re-test reliability [21].

*Albany Panic and Phobia Questionnaire*, *Social Phobia Subscale (APPQSP)*. The APPQSP measures fear in 10 social situations on a scale of 0–8. Scores range from 0–80 with higher scores indicating higher levels of social anxiety. The scale has been shown to have good internal consistency and test-retest reliability [27] and had good internal consistency within this sample (α = .87). Participants showed a wide range of total scores (1–49, *M* = 17.13, *SD* = 11.03).

Participants who were selected for entry into the study also completed:

*Social Behaviour Questionnaire (SBQ)*. Before beginning the experiment, participants completed the SBQ [10] to evaluate how often they used safety behaviours in everyday social interactions. The SBQ has been introduced in Study 1. This sample showed an excellent internal consistency, α = .90.

*Mood Thermometer*. Participants were asked about their current anxiety levels before starting the experiment. To calibrate their responses, they were first asked to rate their anxiety levels for a recent social occasion where they were anxious, and for a different social situation where they were not anxious. This was rated on a 0–100 scale.

*Post-conversation Questionnaire*. Following each conversation, participants were asked to complete a post-conversation questionnaire. This included ratings of how anxious they felt, how anxious they thought they appeared, how anxious they thought their partner looked, how much they liked their partner, how much they enjoyed the conversation, and the extent to which they were self or externally focused. For those who performed the safety behaviours, they also rated how much of the time they used the safety behaviours. All items were rated on a 0–100 scale. After the second conversation, participants were also asked to rate for each conversation how much they would like to have a conversation with their partner again if they both behaved in the same way.

*Assessor’s Behaviour Checklist*. The videotaped conversations were assessed by an independent assessor using a modified version of Stopa and Clark’s behaviours checklist [2]. This comprises 16 items of which eight are positive descriptors (e.g. witty, socially skilled, confident), and eight are negative descriptors (e.g. boring, awkward, anxious). Each is rated on a 0–8 scale (‘not at all’ to ‘extremely’).

#### Procedure

Participants who passed the screening were invited to partake in the experiment. They were given an information sheet and provided written consent for the experiment. Participants were initially kept in separate rooms. The advertising material, information sheet and consent form were ambiguously phrased so that participants believed that both, one, or neither of them might be asked to change some aspect of their behaviour in both, one or neither conversation.

For each safety behaviour subtype we selected three behaviours that were well represented by the avoidance or the impression management factor (see study 1). Participants were asked to perform all three safety behaviours of either the avoidance or impression management type. For avoidance, these were ‘Avoid talking about yourself’, ‘Keep still’ and ‘Talk less’. For impression management, these were ‘Picture how you appear to others’, ‘Check how you’re coming across,’ and ‘Try to come across well’. When they were not required to perform a safety behaviour, participants were instructed to act as they normally would in a conversation.

The conversations were video recorded. The safety behaviour instructions were kept in sealed envelopes, with the experimenter blind to their contents, and given to participants prior to each conversation. The participants had two, five-minute conversations and in between participants were separated and completed the post-conversation questionnaire. After the second conversation, the participants were separated and the post-conversation questionnaire was completed again. The independent assessor later watched and rated the video recordings of the conversations.

#### Statistical analysis

A series of linear mixed models were conducted on the seven self-report measures (self-reported anxiety, self-rating of how anxious one looked, partner-rating of how anxious one looked, enjoyment of conversation, liking by partner, self-focus, desire to repeat conversation) and the independent assessor ratings. The items rated by the independent assessor were summed to create two scores, one for the eight negative descriptors and one for the eight positive descriptors. In total, there were nine outcome variables. The linear mixed models tested the fixed effects of the main effects of conversation type (conversation during which no versus any of the two safety behaviours was performed), participant type (experimental participant, who performed the safety behaviour, versus control participant, who did not), safety behaviour type (avoidance versus impression management safety behaviour) and their two- and three-way interactions. Three levels were specified in the linear mixed models to take into account that the repeated measure factor conversation type was nested within participants and participants were nested in conversation dyads. All three factors were categorical. Baseline anxiety levels and baseline use of safety behaviours were included in the analyses to control for any differences prior to the experiment. Intercepts were specified as random, the covariance structure matrix was unstructured, and Maximum Likelihood was used for model estimation.

### Results

#### Ability to follow safety behaviour instruction

In order to check compliance with the experimental instructions, participants who were instructed to perform safety behaviours were asked to rate the percentage of time that they felt they followed the safety behaviour instruction during the relevant conversation (*M* = 64.12%, *SD* = 13.85%). As all participants indicated that they followed the instructions at least 40% of the time, the data from all of the dyads was used in the analyses. An independent samples t-test also revealed that the degree to which participants felt they could follow the instructions did not vary between avoidance and impression management subgroups, *t*(46) = .42, *p* = .674 (Avoidance–*M* = 64.58, *SD* = 13.59; Impression management–*M* = 64.09 *SD* = 13.68).

#### Participant characteristics

Checks were carried out to examine whether any participant characteristics differed between the individuals who were randomly allocated to the experimental or control conditions and between safety behaviour subtypes (avoidance or impression management). There were no gender differences between the four groups, (χ^2^ (1, *N* = 96) = .38, *p* = .536). Continuous variables (BDI, APPQSP, SBQ, age and pre-experiment anxiety rating) were analysed using a two-way (participant by safety behaviour) multivariate analysis of variance (MANOVA). There was no significant main effect of safety behaviour subtype (*F*(6,41) = .55, *p* = .767, *η*^*2*^ = .07) and no significant participant by safety behaviour interaction (*F*(6,41) = .212, *p* = .971, *η*^*2*^ = .03). However, there was a significant main effect of participant type (*F*(6,41) = 2.36, *p* = .048, *η*^*2*^ = .26). Univariate tests indicated that experimental participants scored higher than control participants on the SBQ (F(1,46) = 5.26, *p* = .026, *η*^*2*^ = .103, experimental–*M* = 28.23, *SD* = 10.49, control–*M* = 22.83, *SD* = 11.60) and anxiety before the start of the conversations (F(1,46) = 8.31, *p =* .006, *η*^*2*^ = .15, experimental–*M* = 25.44, *SD* = 19.09, control–*M* = 15.67, *SD* = 14.78). In view of these differences, in all subsequent analyses anxiety before the conversation and SBQ scores were included to control for any pre-existing differences.

#### Effects of performing safety behaviour sub-types on oneself and the other participant in the conversation

Table 5 shows the descriptive statistics for the measures included in these analyses.

**Table 5:** Effects of safety behaviour subtypes on self and others.

	Avoidance				Impression Management			
	Experimental		Control		Experimental		Control	
	SB *M(SD)*	NSB *M(SD)*	SB *M(SD)*	NSB *M(SD)*	SB *M(SD)*	NSB *M(SD)*	SB *M(SD)*	NSB *M(SD)*
Self-reported anxiety	34.04 (20.88)	20.42 (18.52)	20.83 (15.08)	17.71 (19.84)	21.96 (17.86)	17.42 (21.91)	11.08 (9.68)	13.42 (14.17)
Self-rating of how anxious one looked	55.42 (21.62)	27.92(19.77)	21.75 (19.51)	20.00 (20.75)	20.42 (16.74)	18.46 (18.55)	12.13 (12.03)	14.67 (10.50)
Partner-rating of how anxious one looked	33.75 (22.95)	15.00 (15.25)	25.75 (18.05)	23.04 (20.15)	14.71 (10.31)	13.08 (13.91)	19.50 (16.32)	17.71 (18.60)
Enjoyment of conversation	48.54 (25.51)	74.58 (15.10)	53.46 (22.85)	72.71 (15.53)	60.83 (24.74)	77.92 (13.75)	73.83 (19.89)	71.96 (21.71)
Liking by partner	64.67 (19.23)	76.46 (10.68)	77.29 (11.72)	76.04 (13.99)	80.42 (13.59)	76.25 (17.65)	81.67 (16.53)	79.38 (13.64)
Self-focus	47.08 (24.23)	56.25 (22.47)	61.25 (21.33)	62.29 (22.74)	56.46 (18.74)	54.79 (20.82)	47.71 (29.15)	49.58 (25.15)
Desire to repeat conversation	45.50 (28.12)	72.92 (19.78)	47.58 (24.01)	74.79 (29.02)	80.21 (14.78)	81.88 (14.28)	75.71 (21.62)	77.38 (18.56)
Independent assessor negative ratings	24.21 (8.88)	16.92 (5.21)	19.83 (5.62)	16.33 (6.06)	15.75 (10.04)	14.75 (7.74)	17.50 (5.76)	14.79 (7.87)
Independent assessor positive ratings	32.58 (12.24)	42.29 (9.80)	39.88 (5.75)	42.21 (8.34)	45.79 (11.40)	47.04 (8.07)	44.25 (6.66)	46.79 (9.71)

Note. N = 24 for each of the four groups. B = conversation in which behaviour was performed, NB = conversation in which no behaviour was performed, M = mean, SD = standard deviation.

**Fig 2:**
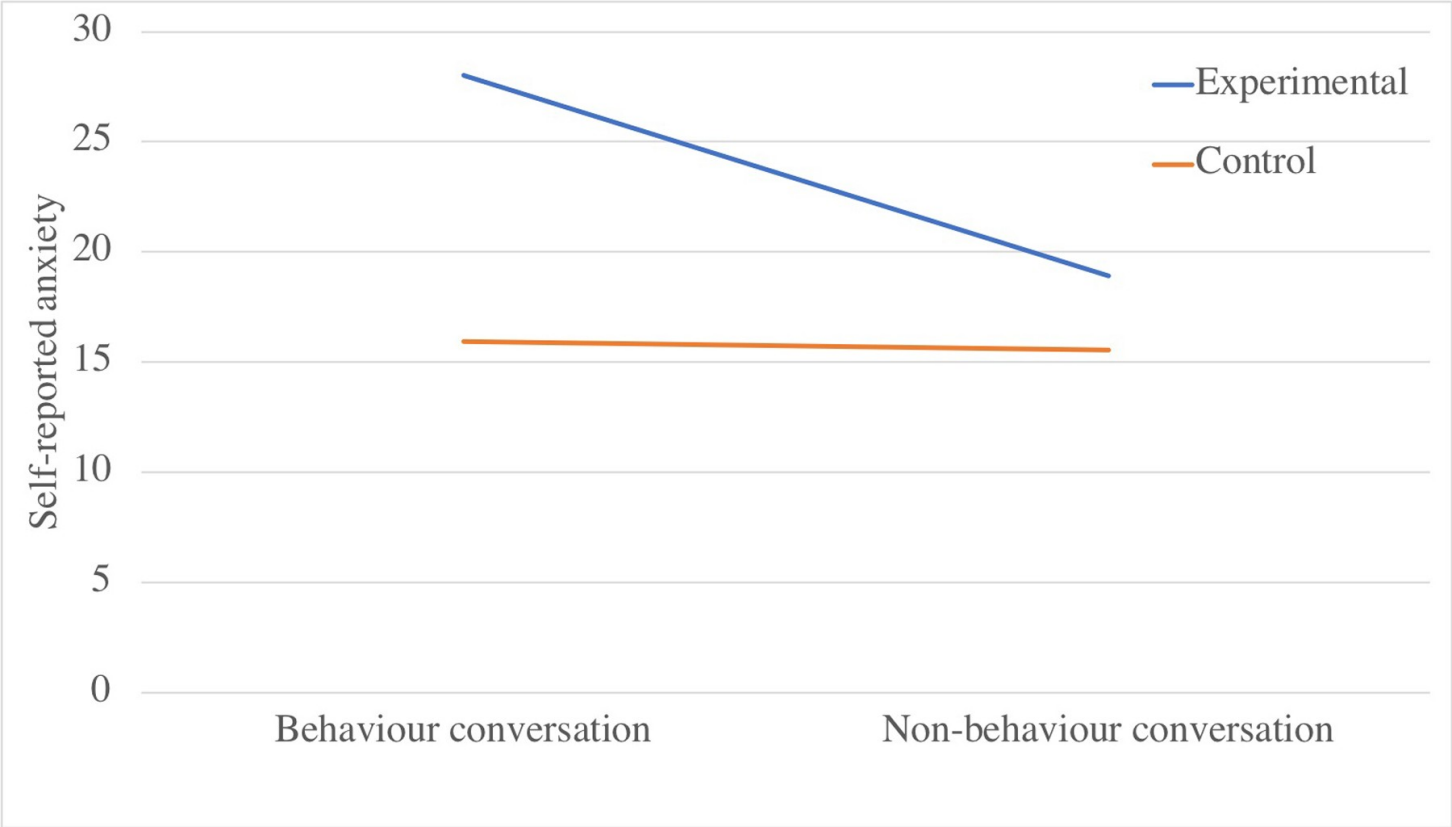
Interaction effect of participant type and conversation type on self-reported anxiety.

*Self-reported anxiety* showed a significant two-way interaction between conversation and participant type, *b* = 10.50, *SE* = 4.61, *p* = .025. Compared to conversations when they were not performing safety behaviours, experimental participants reported feeling more anxious in conversations where they were engaging in safety behaviours (*b* = 9.08, *SE* = 2.93, *p* = .003). For control participants, self-reported anxiety did not differ between the two conversations (*b* = 0.40, *SE* = 1.59, *p* = .805). Fig 2 shows this interaction.

**Fig 3:**
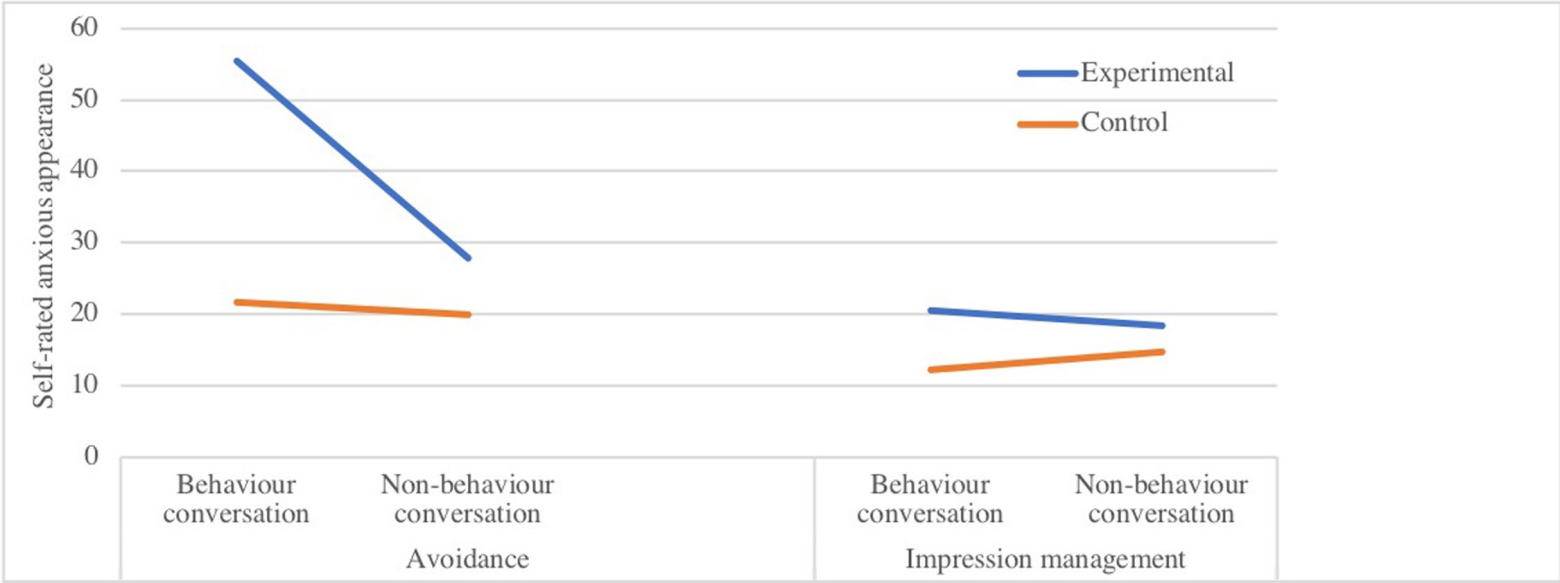
Three-way interaction of safety behaviour type, conversation type and participant type on self-ratings of how anxious one looked.

*Self-rated anxious appearance* showed a two-way interaction between conversation and participant type, (*b* = 25.75, *SE* = 4.83, *p* < .001), which was qualified by a significant three-way interaction (*b* = -21.25, *SE* = 6.82, *p* = .003 (see Fig 3). Further linear mixed models were computed to explore this three-way interaction effect. In the avoidance condition, experimental participants estimated that they looked more anxious when engaging in avoidance safety behaviours than when not doing so (*b* = 25.75, *SE* = 5.87, *p <* .001). For control participants, ratings of how anxious they thought they looked were low and did not differ between the two conversations. In the impression management condition, experimental participants ratings of how anxious they thought they looked did not differ between the conversation in which they did, or did not, engage in impression management safety behaviours. Control participants ratings also did not differ between these two conversations.

*Partner-rating of how anxious one looked* showed a significant two-way interaction between conversation and behaviour type, *b* = 16.04, *SE* = 4.57, *p* = .001, which was qualified by a significant three-way interaction, *b* = -16.21, *SE* = 6.46, *p* = .014. To explore this interaction, separate models were computed for the avoidance and impression management conditions.

Experimental participants appeared more anxious to their partner when engaging in avoidance safety behaviours than when not doing so (*b* = 18.75, *SE* = 3.43, *p* < .001). For control participants, these ratings did not differ between the two conversations (*b* = 2.71, *SE* = 4.01, *p* = .506). There were no significant effects in the impression management condition, indicating that performing impression management safety behaviours during the conversation did not influence one’s partners’ ratings of how anxious one looked.

*Enjoyment of conversation* showed a significant main effect of conversation type (*b* = -19.25, *SE* = 4.22, *p* < .001) and a significant two-way interaction between conversation type and safety behaviour type (*b* = 21.13, *SE* = 5.97, *p* = .001). Both participants enjoyed the conversation less when avoidance safety behaviours were being performed vs not performed (*b* = -19.25, *SE* = 4.58, *p* < .001) but there was no difference in enjoyment when impression management behaviours were, or were not, being performed.

**Fig 4:**
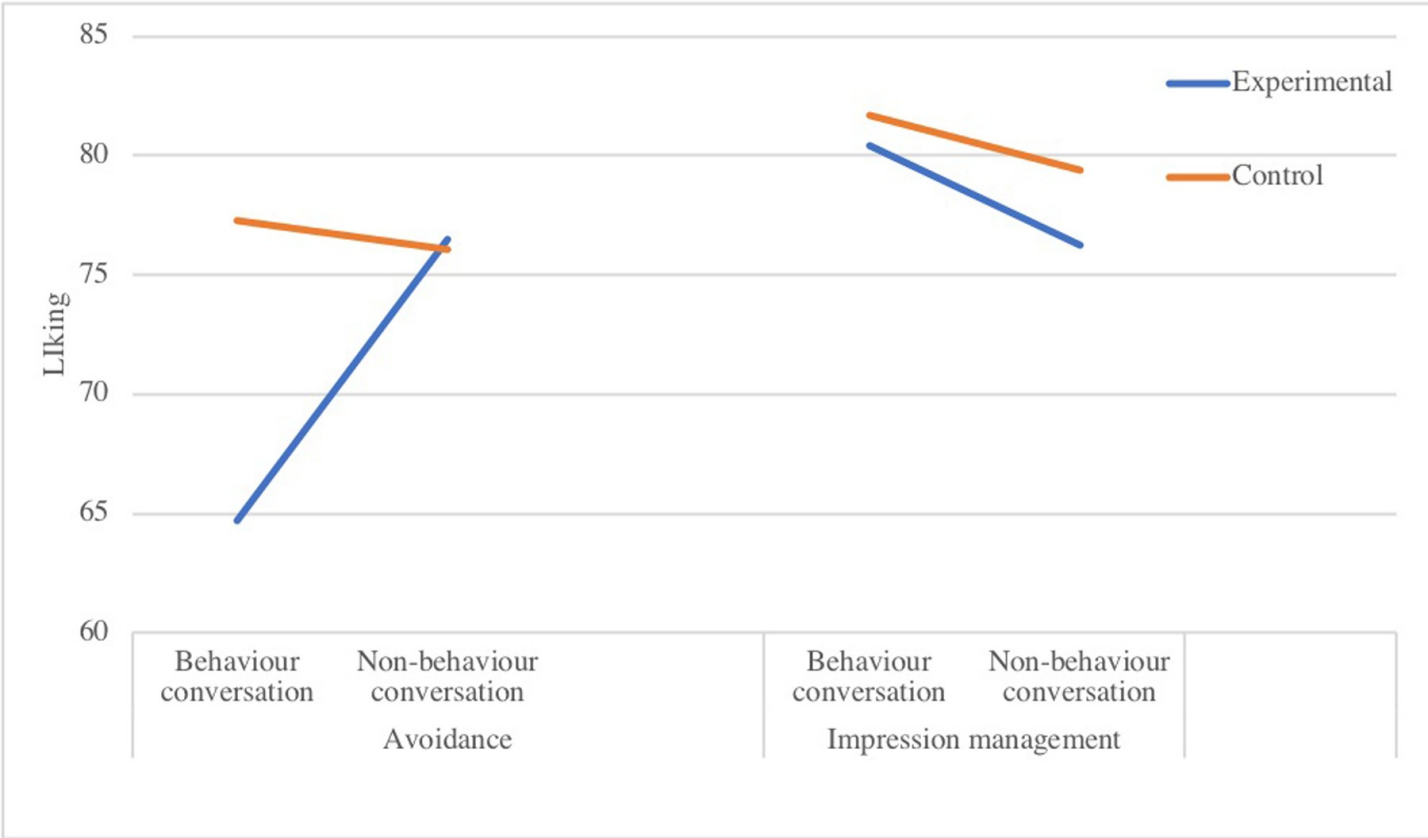
Three-way interaction of safety behaviour type, conversation type and participant type on how much one is liked by one’s partner.

*Liking by partner* showed a main effect for conversation type (*b* = -11.79, *SE* = 2.66, *p* < .001) and significant two-way interactions between conversation and participant type (*b* = 13.04, *SE* = 3.76, *p* = .001), and between conversation and safety behaviour type (*b* = 15.96, *SE* = 3.76, *p* < .001). These effects were qualified by a significant three-way interaction (see Fig 4) on how much the conversation partners liked each other, *b* = -14.92, *SE* = 5.31, *p* = .006.

To explore this interaction, separate models were computed for the avoidance and impression management conditions. Experimental participants were liked less by their partners when they engaged in avoidance safety behaviours than when they were not doing so (*b* = -11.79, *SE* = 2.82, *p* < .001). Control participants were liked equally in both conditions (*b* = 1.25, *SE* = 2.82, *p* = .662). There were no significant differences in the impression management condition, indicating that performing impression management safety behaviours did not have an adverse effect on how much one is liked by one’s partner.

*Self-focus* showed no significant main effects or interactions.

**Fig 5:**
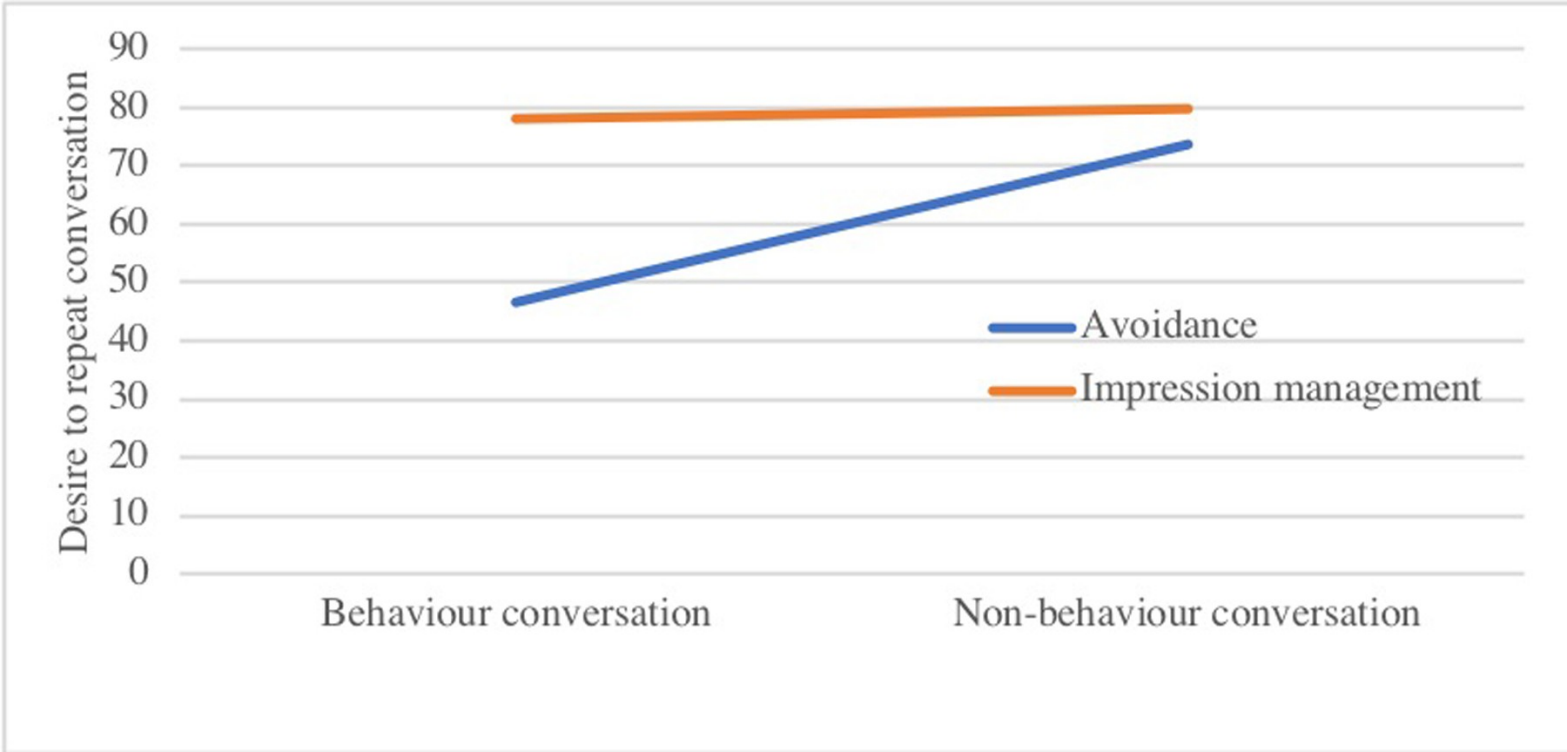
Interaction effect of safety behaviour type and conversation type on desire to repeat conversation.

*Desire to repeat conversation* showed a significant main effect of conversation type (*b* = -27.21, *SE* = 4.89, *p* < .001) which was qualified by a two-way interaction between conversation and safety behaviour type, *b* = 25.54, *SE* = 8.92, *p* < .001 (see Fig 5): Both participants wished to repeat the conversation less when avoidance behaviours were performed during the conversation compared to not performing such safety behaviours. There were no significant differences in the impression management condition.

*Independent assessor positive ratings* produced a significant two-way interaction between conversation and participant type (*b* = -7.38, *SE* = 2.40, *p* = .003) which was qualified by a significant three-way interaction effect (*b* = 8.67, *SE* = 3.40, *p* = .012). This three-way interaction effect was further explored by computing separate models for the avoidance and impression management safety behaviour conditions and for their direct comparison. The experimental participant was rated less positively when engaging in avoidance safety behaviours compared to when not doing so (*b* = -9.71, *SE* = 2.64, *p* = .001) and compared to when performing impression management behaviours (*b* = 8.88, *SE* = 3.02, *p* = .005). There were no significant differences in assessor ratings of the control participant.

*Independent assessor negative ratings* produced a significant main effect of conversation type (*b* = 3.50, *SE* = 1.42, *p* = .016). The behaviour conversations were rated more negatively than the non-behaviour conversations. There were no other significant main effects or interaction effects. However, there was a marginally significant three-way interaction on independent assessor negative ratings (*b* = -5.50, *SE* = 2.84, *p* = .056), which mirrored the three-way interaction for positive ratings.

### Discussion

The results of Study 2 indicate that avoidance and impression management behaviours have non-identical effects on conversations. Both safety behaviours make the person who performs them feel more anxious. It is therefore clear that both play a causal role in generating feelings of social anxiety. This is the exact opposite effect to that intended by individuals who use them. In general, socially anxious individuals use safety behaviours to try to manage a social situation but our results, which are in line with theoretical models [9, 28], indicated that when considering how anxious someone feels, performing either safety behaviour makes things worse.

When one looks at the dependant variables other than feeling anxious, there is evidence that the two types of safety behaviour have different effects. There is no variable for which performing the safety behaviour produces a more positive outcome than not performing the safety behaviour. However, for many variables the harmful effects are more marked with avoidance safety behaviours than with impression management safety behaviours. In particular, while performing avoidance safety behaviours, but not while performing impression management safety behaviours, participants thought that they looked more anxious, enjoyed the conversation less, and were less keen on having a further conversation. There were also negative effects of avoidance safety behaviours on the other person in the conversation. If participants performed avoidance safety behaviours the person they were talking with rated them as appearing more anxious and liked them less. They also enjoyed the conversation less and were less keen on having a further conversation with a partner who was performing avoidance safety behaviours. Independent assessor ratings showed a similar pattern. When participants performed avoidance safety behaviours the independent assessor gave them lower ratings on the positive items of the conversation checklist. This effect was not observed with impression management safety behaviours.

These experimental results are in line with the correlational findings reported by Plasencia et al. [14] and support the view that both types of safety behaviours have a causal role in increasing subjective anxiety but only the avoidance sub-type has a clear-cut negative effect on others.

The results of this study have implications for the treatment of SAD. Both types of safety behaviours have the opposite effect to the one that was intended. Patients use them in an attempt to decrease anxiety, but they consistently increase anxiety. Demonstrating this in therapy is therefore likely to be a helpful strategy for enabling patients to start to drop their unhelpful safety behaviours. Cognitive therapy for SAD includes a behavioural experiment that facilitates this process by encouraging patients to have a social interaction with and without safety behaviours and comparing how they feel in the two conditions [e.g. 4, 29]. Furthermore, this study shows the need to look closely at the types of safety behaviours that patients are using and formulate treatment strategies accordingly.

As avoidance safety behaviours have further adverse effects on the people that socially anxious individuals interact with, it is likely to be helpful to include in therapy a focus on such adverse effects and how to reduce them. Insight into the way that avoidance safety behaviours might be perceived by other people can be achieved through activities such as a reverse role play, wherein the therapist performs avoidance behaviours during a conversation [e.g. 30, 31] or by using video-feedback [32]. In either case, the aim of the procedure is help the patient understand that safety behaviours are conveying to other people the opposite impression to the one that the patient would like to convey. In particular, the patient is likely to want to be liked and accepted by other people, but the avoidance safety behaviours are likely to convey the impression that the patient is not interested in talking to other people and perhaps doesn’t like them. Insight into this effect can be used to help patients experiment with dropping their avoidance safety behaviours and discover that they will often be accepted by others once they make it clear that they are interested having an interaction.

### Limitations

Study 2 was designed to test the causal role of avoidant and impression management safety behaviours by manipulating them and observing their effects in a non-clinical population. While the findings are consistent with the causal hypothesis, they should be confirmed by repeating the study with patients who meet diagnostic criteria for social anxiety disorder. We argued that if safety behaviours have a causal role in generating anxiety in social interactions that effect should also be evident if non-clinical individuals use the safety behaviours. However, non-clinical individuals would be less likely to use the safety behaviours in everyday life and it is possible this makes a difference. In addition, when safety behaviours are used naturalistically they are deployed for a particular purpose. This type of motivated use cannot easily be modelled in an experimental study.

The study could have benefited from a further active control condition in which participants followed a set of conversation instructions that may have been similarly absorbing as the safety behaviours but were not safety behaviours. This would have clarified the extent to which any adverse effects of safety behaviours are due to task distraction or go beyond the effects of that process.

The experimental and control participants were matched on most baseline variables, including a well-validated measure of social anxiety (APPQSP). However, despite random allocation to the two conditions, there were some baseline differences on the SBQ and the mood thermometer. These were statistically controlled by using the variables as covariates. Whilst this is a commonly accepted practice, it has been criticised by some authors [33].

Finally, our non-clinical population scored higher on impression management safety behaviours than avoidance safety behaviours, suggesting that they were more familiar with the latter. It is possible that less familiar impression management strategies might have produced different results.

## Conclusion

In conclusion, these two studies help add to our understanding of the use and the effect of different safety behaviours in social anxiety disorder. Study 1 showed that the use of social safety behaviours is higher in individuals with SAD than those with PTSD or non-patient controls. This was not due to higher levels of general anxiety or depression. The factor analysis performed on individuals with SAD produced two factors, termed avoidance and impression management, as rationally grouped by Hirsch et al. [3] and found by Plasencia et al. [14]. This adds support to the existence of these sub-types of safety behaviour in SAD. Study 2 suggests that these two sub-types have different effects on conversational outcomes, with avoidance having a more globally negative effect. Further research into this division of safety behaviours will help to confirm and clarify this distinction, leading to more effective and specialised treatment for individuals suffering from SAD.
